# Validation of a primer optimisation matrix to improve the performance of reverse transcription – quantitative real-time PCR assays

**DOI:** 10.1186/1756-0500-2-112

**Published:** 2009-06-23

**Authors:** Thomas Mikeska, Alexander Dobrovic

**Affiliations:** 1 Molecular Pathology Research and Development Laboratory, Department of Pathology, Peter MacCallum Cancer Centre, Locked Bag 1, A'Beckett Street, Melbourne, Victoria 8006, Australia; 2 Department of Pathology, University of Melbourne, Parkville, Victoria 3010, Australia

## Abstract

**Background:**

The development of reverse transcription – quantitative real-time PCR (RT-qPCR) platforms that can simultaneously measure the expression of multiple genes is dependent on robust assays that function under identical thermal cycling conditions. The use of a primer optimisation matrix to improve the performance of RT-qPCR assays is often recommended in technical bulletins and manuals. Despite this recommendation, a comprehensive introduction to and evaluation of this approach has been absent from the literature. Therefore, we investigated the impact of varying the primer concentration, leaving all the other reaction conditions unchanged, on a large number of RT-qPCR assays which in this case were designed to be monitored using hydrolysis probes from the Universal Probe Library (UPL) library.

**Findings:**

Optimal RT-qPCR conditions were determined for 60 newly designed assays. The calculated C_q _(Quantification Cycle) difference, non-specific amplification, and primer dimer formation for a given assay was often dependent on primer concentration. The chosen conditions were further optimised by testing two different probe concentrations. Varying the primer concentrations had a greater effect on the performance of a RT-qPCR assay than varying the probe concentrations.

**Conclusion:**

Primer optimisation is important for improving the performance of RT-qPCR assays monitored by UPL probes. This approach would also be beneficial to the performance of other RT-qPCR assays such as those using other types of probes or fluorescent intercalating dyes.

## Background

The need for gene expression platforms that can simultaneously assay multiple gene transcripts from routine pathological biopsies is increasing. Microarrays are not the ideal solution as they suffer from poor dynamic range and the need for high quality material. In particular, high quality material is often not available such as when formalin-fixed paraffin-embedded (FFPE) sections are being used.

Reverse transcription – quantitative real-time PCR (RT-qPCR) is the preferred method to quantify RNA when a wide dynamic range and high signal to noise ratios are desired. RT-qPCR involving a multiple gene transcript panel needs to be custom-designed to provide the most flexibility in gene transcript selection.

A robust, reproducible, and optimised RT-qPCR assay is one of the key requirements for reliable gene expression data. Running an RT-qPCR under suboptimal conditions results in higher variability between replicates [1] and may also result in decreased sensitivity [2]. Unfortunately, RT-qPCR optimisation has become disregarded by many research groups in the era of high throughput analysis and rapid data reporting [2].

In order to set up multiple RT-qPCR assays for gene expression profiling it is necessary to run them at common thermal cycling parameters, which precludes assay optimisation by varying the annealing temperature. An effective way to optimise RT-qPCR assays, and enabling the use of common PCR conditions is to vary the primer concentrations [3]. This can also compensate for small errors in the calculation of the effective primer melting temperature [4,5].

RT-qPCR analysis is a multi-step process. The quality and quantity of the starting material, as well as each step, will contribute to the success of a result. Therefore, each step needs careful handling to ensure accurate results [2,6,7] and should be reported in a standardised format like outlined in the recently released MIQE (Minimum Information for Publication of Quantitative Real-Time PCR Experiments) guidelines [8].

RT-qPCR assays have been based either on fluorescent, non-sequence specific intercalating reporter dyes or sequence specific fluorescent probes [9]. As intercalating dyes bind non-specifically to double stranded DNA generated during the PCR reaction, the introduction of an internal amplicon probe improves specificity by eliminating noise from non-specific amplification such as primer dimers.

Whereas the principles of good primer design have been stressed for those performing RT-qPCR assays using an intercalating fluorescent dye, the use of probe-based assays has often led to the belief that the specificity of the probe means that less care is necessary with primer design and assay optimisation [10].

The Universal Probe Library (UPL) platform [11] uses gene-specific primers in combination with a library of hydrolysis probes, dually labelled with fluorescein (FAM) and a proprietary dark quencher. A web based program facilitates the choice of primers to enable the use of one of the hydrolysis probes to establish a RT-qPCR assay for almost any gene transcript. The amplicons are generally very short allowing the analysis of degraded RNA such as that obtained from FFPE tissues.

In this communication, we have validated the impact of using a primer optimisation matrix and probe concentration optimisation on the performance of a large panel of newly designed RT-qPCR assays to profile the expression pattern of human DNA repair gene transcripts. First, the best performing primer concentrations for a given assay were determined. The optimum probe concentration for the optimal primer concentrations was then identified.

## Results and Discussion

Three different primer concentrations, 100 nmol/L, 200 nmol/L (the recommended starting concentration for UPL assays), and 300 nmol/L, and their combinations in a primer optimisation matrix were investigated, leaving all the other reaction conditions unchanged. A primer combination was considered to be optimal when the amplification resulted in an amplicon of the expected size where the following conditions were met; a low C_q _value (the point where fluorescence intensity during amplification is significantly greater than background fluorescence), a low standard deviation between replicates, adequate signal to noise ratio in the sense that robust levels of fluorescence intensity were seen, and no (or very low levels of) primer dimers were present.

The RT-qPCR with the optimal primer concentration combination was further optimised with respect to the concentration of the probe. In preliminary experiments, a wider range of UPL probe concentrations were tested. Probe concentrations higher than 200 nmol/L did not significantly improve RT-qPCR assays. Furthermore, the cost of a probe-based RT-qPCR assay is strongly dependent on the cost of the probe. We therefore only used the concentrations 100 nmol/L and 200 nmol/L for further probe optimisation. The lower probe concentration was chosen except when the higher probe concentration gave a significantly lowered C_q _value and/or resulted in a significant improvement of the signal intensity (signal to noise ratio).

We designed 63 RT-qPCR assays for a panel of DNA repair and reference genes using the web-based design service for UPL RT-qPCR assays as outlined in Material and Methods. An intron-spanning assay was not possible for eight (13%) of the genes. Two of these genes consisted of a single exon, whereas the software was only able to choose intra-exonic primers for the remaining six.

Sixty assays (95%) were successful after the use of the primer matrix to optimise the concentration of each primer. Three assays, did not meet the quality control criteria as they either gave multiple non-specific bands as seen on an agarose gel or gave poor amplification with all primer concentrations tested.

**Figure 1:**
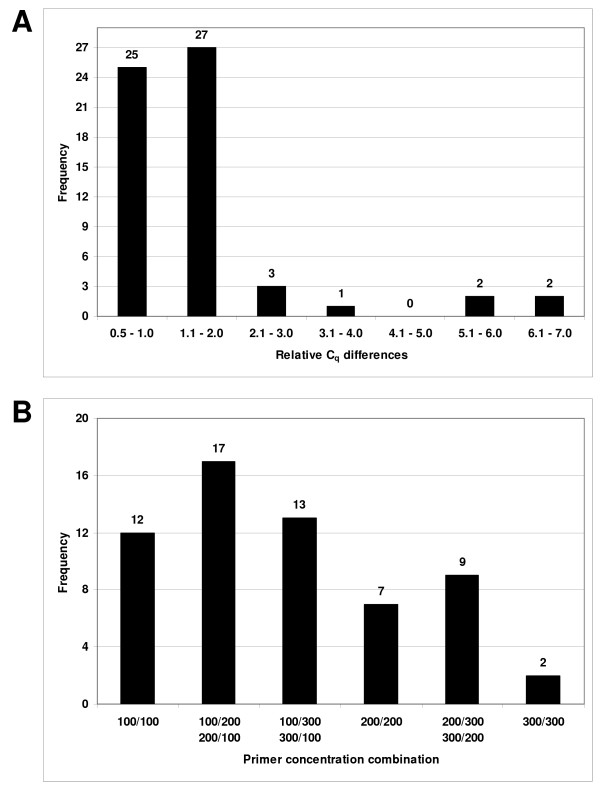
**Determining the optimum primer concentrations**. (A) Distribution of the relative C_q _differences (the difference between the primer combination showing the highest C_q _value and the primer combination showing the lowest C_q _value for each assay) obtained for each of the 60 RT-qPCR assays. (B) Distribution of the optimal primer concentration combinations for each of the 60 assays. The primer concentration combinations are given in nmol/L for the forward and the reverse primers respectively.

The C_q _differences (primer combination showing the highest C_q _value minus primer combination showing the lowest C_q _value for a given RT-qPCR assay) observed for the 60 assays were in the range of 0.5 to 6.7. The observed C_q _differences for a given RT-qPCR assay are due to the varying primer concentrations, as these were the only variable reaction parameters. The performance of the majority of the RT-qPCR assays were significantly dependent on primer concentration (Fig. 1A). Twenty seven assays (45%) had C_q _value differences in the range of 1.1 to 2.0, and eight assays (13%) had a C_q _value difference greater than 2.1. Twenty five assays (42%) that had C_q _value differences in the range of 0.5 to 1.0 and were therefore less dependent on primer concentration.

The RT-qPCR products were also examined by gel electrophoresis before the optimal primer concentration combination was chosen to eliminate any conditions that gave unacceptable amounts of primer dimers.

The distribution of optimal primer concentrations is shown in Figure 1B. Thirty nine assays (65%) performed better with an asymmetric primer concentration combination, while symmetric primer concentrations performed better in 21 assays (35%).

**Figure 2:**
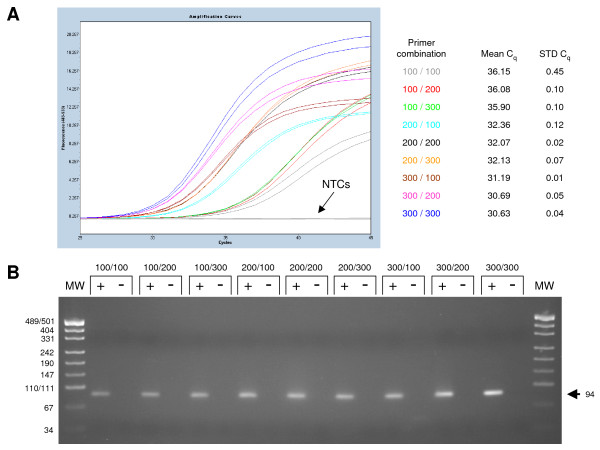
**RT-qPCR optimisation of the *NBS1 *gene**. (A) Amplification curves for the nine primer concentration combinations of the primer optimisation matrix. No amplification was seen for the no template controls (NTCs). The primer concentration combinations are indicated in the same colour as the corresponding curves. The mean C_q _values and standard deviations (STD) were calculated from the amplification curves as outlined in Material and Methods. The primer concentration combinations are given in nmol/L for the forward and the reverse primer, respectively. (B) Digital image of *NBS1 *RT-qPCR reactions obtained for the primer optimisation matrix. Quality control of the RT-qPCR reactions by gel electrophoresis revealed only the *NBS1 *amplicon without additional, non-specific bands. The sizes of the molecular weight markers (MW) are given on the left, whereas the size of the *NBS1 *amplicon is indicated on the right (arrow). Wells loaded with RT-qPCR reactions are labelled with (+), while the respective no template control is labelled with (-). Sizes are given in base pairs and primer concentration combinations are given in nmol/L respectively.

As an example, the C_q _values of the RT-qPCR assay for the *NBS1 *gene using different primer concentrations showed a difference of 5.5 (Fig. 2A). Each of the primer concentration combinations generated a specific amplicon as shown by gel electrophoresis (Fig. 2B) but 300 nmol/L of each primer performed best showing the lowest C_q _value (Fig. 2A). In this case, the concentration of the forward primer had a greater contribution to higher RT-qPCR sensitivity than the concentration of the reverse primer (as seen in Fig. 2A).

After the optimal primer combination was chosen, we optimised the concentration of the probe for that particular primer combination. Forty seven of 60 assays (78%) were found to be optimal with 100 nmol/L, whereas 13 assays (22%) performed better with a probe concentration of 200 nmol/L.

When a default concentration of 200 nmol/L for each primer was used, a satisfactory result was observed in 54 out of 63 assays (86%). Nevertheless, only seven assays out of 60 (12%) performed best using a primer combination of 200 nmol/L for each primer (Fig. 1B). This makes it clear that optimising a new RT-qPCR assay is essential to guarantee its efficiency as well as its specificity.

The range of concentrations to test in the primer optimisation matrix as well as the concentrations of the probe to use are likely to be dependent on the amplification monitoring system used and needs to be determined for each system separately.

## Conclusion

In order to set up multiple RT-qPCR assays for gene expression profiling it is necessary to run them at common thermal cycling parameters, thereby precluding assay optimisation by varying the annealing temperature. We have shown that the use of the primer optimisation matrix in combination with gel electrophoresis of RT-qPCR reactions is important for the development of each component RT-qPCR assay. The optimisation of the probe concentration may further improve the sensitivity as well as the signal-noise ratio for some assays. Developing RT-qPCR assays generating only specific amplicons also opens up the possibility of using probe independent assays. Therefore, we recommend RT-qPCR optimisation should be routinely performed for each new assay in the laboratory.

## Materials and methods

### RNA extraction and complementary DNA (cDNA) preparation

The HL60 cell line and human peripheral blood mononuclear cells from normal healthy volunteers were used as different sources for total RNA. Total cellular RNA was immediately extracted from harvested cells using TRIzol reagent (Invitrogen, Carlsbad, CA) according to the protocol of the supplier and quantified with a Nano-Drop ND-1000 spectrophotometer (NanoDrop Technologies, Wilmington, DE). RNA purity was estimated by the absorbance ratio A_260_/A_280_. The calculated ratios were in the range of 1.8 to 1.9 for the HL60 cell line samples, and 2.1 for the human peripheral blood mononuclear cell samples and indicate high purity RNAs. mRNA integrity was assessed by the 3':5' assay using the *GAPDH *gene mRNA (NM_002046) as the target sequence [2]. The samples showed 3':5' ratios of 1 to 2, which indicate high quality mRNAs.

One microgram total RNA was reverse transcribed using the Superscript III Reverse Transcriptase (Invitrogen) with 250 ng random hexamer primers (Pharmacia, Uppsala, Sweden) according to the manufacturer's instructions, without an RNase inhibitor in a final volume of 20 μL. The mixture was incubated for one hour at 50°C.

### Universal Probe Library (UPL) assay design

The target input sequences were chosen to cover transcript information available for splice variants at the Ensembl Genome Browser [12] and the National Center for Biotechnology Information (NCBI). RT-qPCR primers and an appropriate probe were chosen by the UPL Assay Design Center web service for 63 human genes. The program is mainly based on the Primer3 software [13] with additional features (e.g. identification of pseudogenes). The default parameters were used. For each gene, the chosen RT-qPCR assay was the most highly ranked by the design software and belonged to a common assay that covered all splice variants given by the input sequence.

### Reverse transcription – quantitative real-time PCR (RT-qPCR) and gel electrophoresis

PCR was performed on the LightCycler 480 Instrument (Roche Diagnostics, Basel, Switzerland). Resulting data were analysed and quantified with the LightCycler 480 software release 1.5.0 (Roche), utilising the second derivative maximum method [14]. The calculated C_P _(Crossing Point) value is the recommended term C_q _(Quantification Cycle) value [8].

PCR was performed in white LightCycler 480 Multiwell Plate 96 plates (Roche) in a final reaction volume of 10 μL. According to the primer optimisation matrix, varying amounts of the forward and reverse primer (GeneWorks, Adelaide, Australia) of 100 nmol/L, 200 nmol/L, and 300 nmol/L were mixed in 1× LightCycler 480 Probes Master (Roche) containing 100 nmol/L and 200 nmol/L of the human Universal Probe Library probe (Roche), respectively, and 1.0 μL of cDNA as template (1:20, taken from one appropriate source described above, but consistent throughout a single experiment). The initial denaturation (95°C, 10 minutes) was followed by 45 cycles of 10 seconds at 95°C, 30 seconds at 60°C, and a final cooling step at 40°C for 10 seconds. Each primer concentration combination was analysed in duplicate for each cDNA source used, while the no template control was performed only once.

The quality of the RT-qPCR products of each primer concentration combination was evaluated by gel electrophoresis. The samples were run on a 2.5% (w/v) agarose gel in a 1× TBE Buffer system, pH 8.3, at 100 V/cm and stained with ethidium bromide. The wells were loaded with 10 μL of the RT-qPCR reactions mixed with 2.5 μL 5× loading dye. One μL pUC19/*HpaII *DNA Molecular Weight Marker (GeneWorks) was run alongside the PCR products to determine their size.

## Competing interests

The authors declare that they have no competing interests.

## Authors' contributions

TM designed and performed the experiments, analysed the data and wrote the manuscript. AD initiated the project, supervised the work and co-wrote the manuscript. Both authors have read and approved the manuscript.
